# Bread

**Published:** 1870-11

**Authors:** 


### BREAD.

In every hundred bushels of wheat, more than a thousand pounds of valuable nutriment is lost to human use by the present method of grinding wheat into flour, and preparing it for the table.

If you take a grain of wheat, you will find that it contains a hard, thin covering, which is indigestible, and without any nutriment whatever, and in making the white flour out of the whole grain of wheat, this substance is separated, and is known by the name of bran; but this bran has attached to it the most valuable part of the grain of wheat, the part which gives strength to the bones, beauty and durability to the teeth, and vigor to brain and body; but if the outer skin is removed from the grain of wheat before it is ground, then the result before named is attained, and millions of dollars' worth of nutriment is saved to the human family every year.

Machinery has been devised by which every grain of wheat is thus denuded of its external skin, hard, innutritious and indigestible as it is, without allowing any of the nutrient portions of the grain to adhere.

Out of this denuded wheat, two kinds of flour are made: First, family flour, the whitest, used for loaf bread, one hundred and fifty pounds of which are obtained from two hundred and forty pounds of wheat, twenty pounds of which is pure bran, a woody fibre, without an atom of nutriment; but the other seventy pounds is what is excluded in making white family flour, and constitutes the clear loss in every two hundred and forty pounds of wheat. The second kind of flour is the whole product of the skinned or debranned wheat ground, and is called "cerealine," from the word cereal, which means the various grains which makes bread. This cerealine is the residue of the whole berry after the skin has been removed, and is the meat and bread of the "staff of life," and will go nearly "twice as far"

in feeding a family as the ordinary family flour. It is an admirable material for short-cake, muffins, floured and batter cakes, if stirred in water, using yeast for rising—or by employing milk and eggs, no rising is needed. And what is surprising is, it can be sold at the price of common flour, and will give a morning hot cake, which will not only not give dyspepsia, but tends to cure it.

This cerealine is not only more digestible, but is an actual saving of nutriment of seventy parts out of two hundred and forty parts of common flour, and if used by the young, instead of the common family flour, would in time close half the dentistries in the land. The only place known to the editor for obtaining this cerealine, is at Hood's Mills, Carrol County, Maryland. It ought to be sold in New York city, a million dollars' worth a year; but whether there are families enough in the metropolis of the continent to pay a profit on the enterprise, remains to be seen.

